# Supplementary material for: Inhibiting weld cracking in high-strength aluminium alloys
Source: Nat Commun. 2022 Oct 3;13:5816. doi: 10.1038/s41467-022-33188-x (PMC9530225; doi:10.1038/s41467-022-33188-x)
Supplement: Supplementary file 1 — Supplementary Information [file 41467_2022_33188_MOESM1_ESM.pdf]

Supplementary Information for

Inhibiting weld cracking in high-strength aluminium alloys

Yanan Hu [1,2], Shengchuan Wu [1,3,*], Yi Guo [4], Zhao Shen [5,6,*] , Alexander M. Korsunsky [7], Yukuang Yu [1], Xu Zhang [2], Yanan Fu [8], Zhigang Che [9], Tiqiao Xiao [8], Sergio Lozano-Perez [6], Qingxi Yuan [10], Xiangli Zhong [3], Xiaoqin Zeng [5], Guozheng Kang [1,2], Philip J. Withers [3,*]

[1] State Key Laboratory of Traction Power, Southwest Jiaotong University, Chengdu 610031, PR China. [2] School of Mechanics and Aerospace Engineering, Southwest Jiaotong University, Chengdu 611756, PR China. [3] Henry Royce Institute, Department of Materials, The University of Manchester, Manchester M13 9PL, UK. [4] Institute of Metal Research, Chinese Academy of Sciences, Shenyang 110016, PR China. [5] School of Materials Science and Engineering, Shanghai Jiao Tong University, Shanghai 200240, PR China. [6] Department of Materials, University of Oxford, Oxford OX1 3PH, UK. [7] Department of Engineering Science, University of Oxford, Oxford OX1 3PJ, UK. [8] Shanghai Synchrotron Radiation Facility (SSRF), Shanghai Advanced of Sciences, Shanghai 201204, PR China. [9] Science and Technology on Power Beam Processes Laboratory, AVIC Manufacturing Technology Institute, Beijing 100024, PR China. [10] Beijing Synchrotron Radiation Facility (BSRF), Chinese Academy of Sciences, Beijing 100049, PR China.

*e-mail: wusc@swjtu.edu.cn; shenzhao081@sjtu.edu.cn; p.j.withers@manchester.ac.uk.

Supplementary Table 1: Examples of welds from the literature reported as showing FQZs.

Material	Welding process	Application	Reference	FQZ
2000-series Al alloy (2219)	LBW and HLAW	Aviation and aerospace	[1][2]	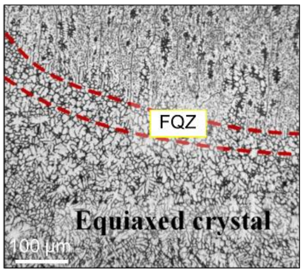
2219 Al alloys	GTAW	Astronautics	Our result	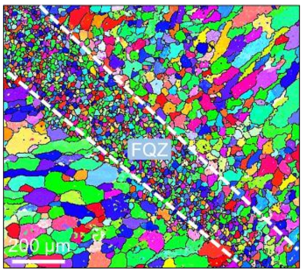
6000-series Al alloy (6061)	HLAW	Aircraft, high-speed train and automobile	[3]	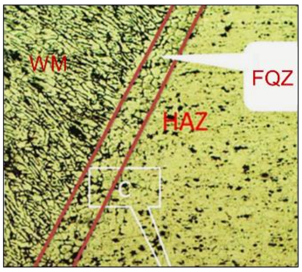
7020 Al alloys	HLAW	High-speed railway vehicle	[4][5]	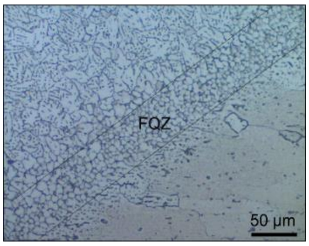
A7N01 alloys	HLAW	High-speed railway vehicle	Our result	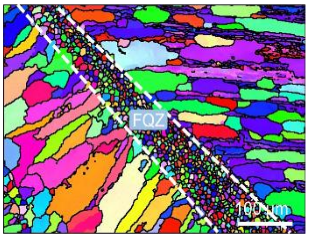

S960 steel	LBW	Pressure vessel	[6]	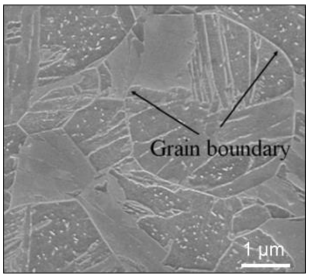
G115 steel	GTAW	High steam boiler tube and power plant	[7]	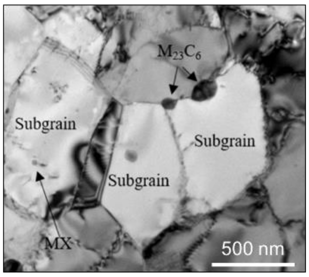
Cr. 92 steel	GTAW	Steam pipe and fossil-fired power plant	[8]	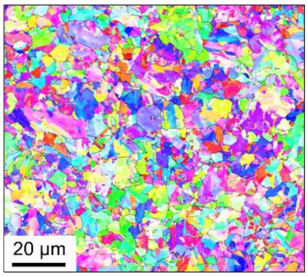

Supplementary Table 2: Estimates of the various strengthening contributions for each weld zone.

Materials	σ_0 (MPa)	$\Delta\sigma_{dis}$ (MPa)	$\Delta\sigma_{ss}$ (MPa)	$\Delta\sigma_{gb}$ (MPa)	$\Delta\sigma_{ppt}$ (MPa)	σ_y^{cal} (MPa)
BM	10	140	96	20	185	451
HAZ	10	141	90	18	144	403
WM	10	137	65	17	117	346
FQZ	10	134	78	45	45	312

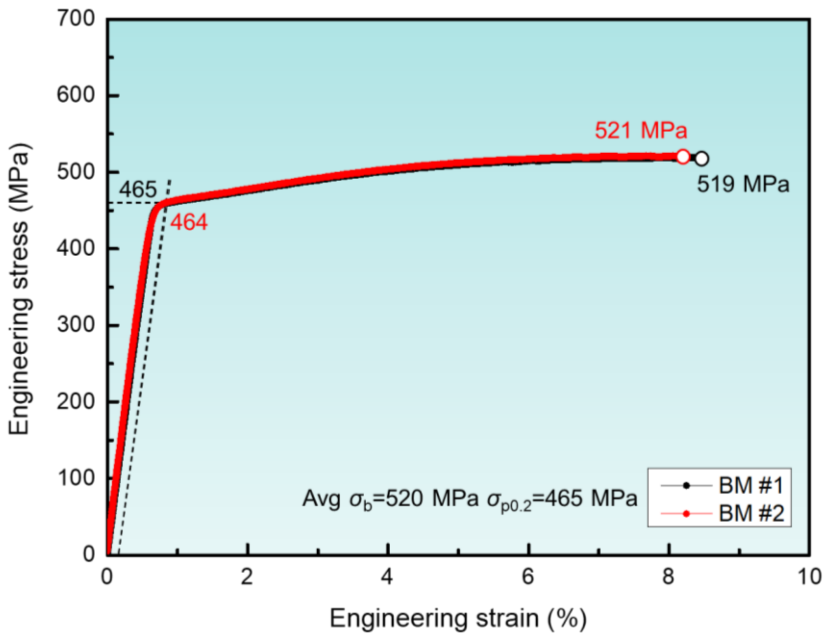

Supplementary Fig. 1: Engineering stress-strain curves of the base materials. Tests for two samples are shown (BM#1 and BM#2) along with their 0.2% yield points (dashed line).

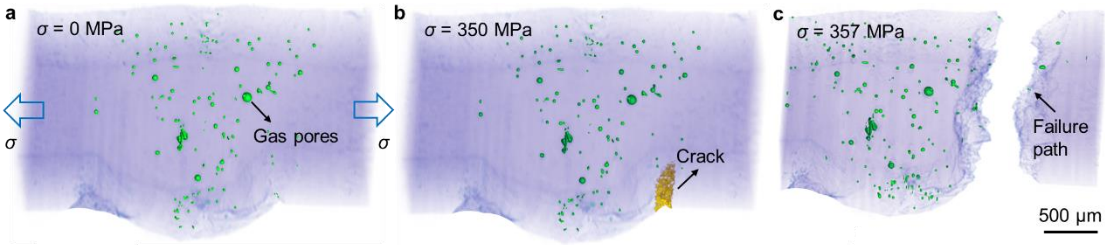

Supplementary Fig. 2: SR-μCT based reconstructed 3D volume renderings (pores rendered green and cracks yellow) of the tensile specimen. a, At a nominal stress of 0 MPa. **b,** 350 MPa. **c,** 357 MPa.

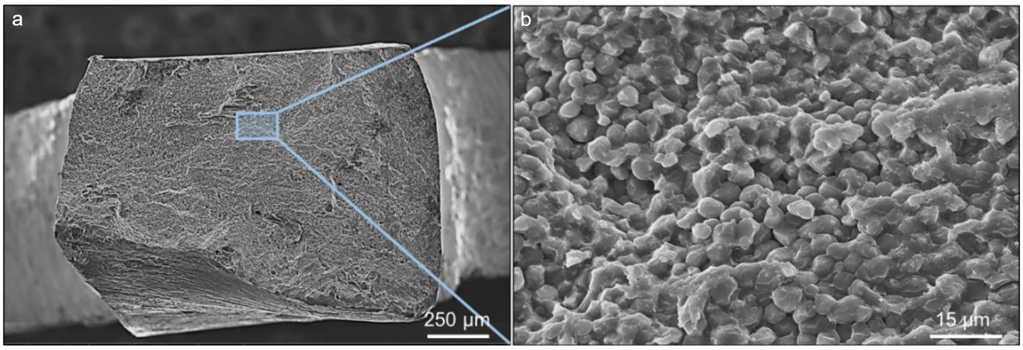

Supplementary Fig. 3: SEM images showing the fractography of a welded joint at different magnifications. a, Lower magnification. **b,** Higher magnification.

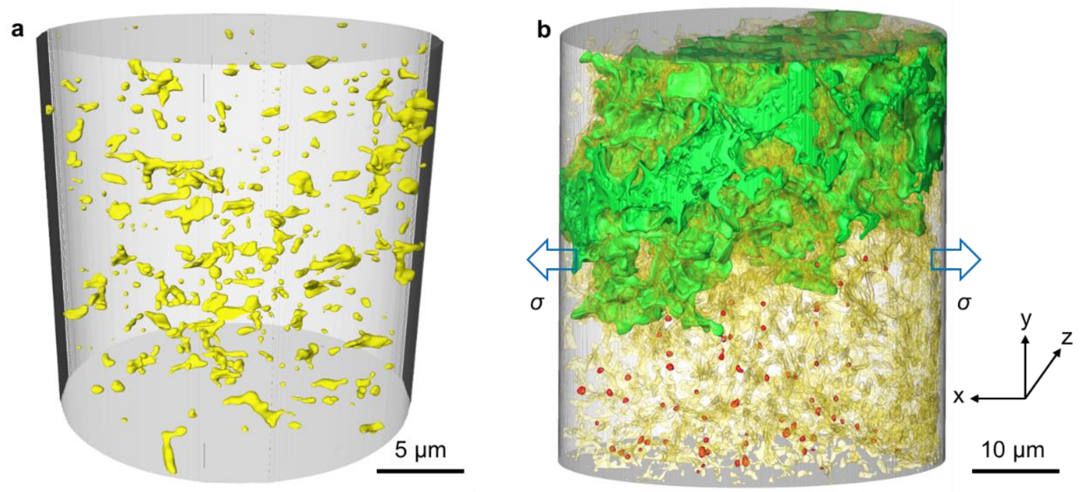

Supplementary Fig. 4: Volume renderings of NanoCT scans for micropillars cut from the FQZ showing the large-sized long-range connected voids (green), the intergranular phases (yellow) and the nucleated micro-voids (red). a, Unstrained FQZ. **b,** Post-strained FQZ.

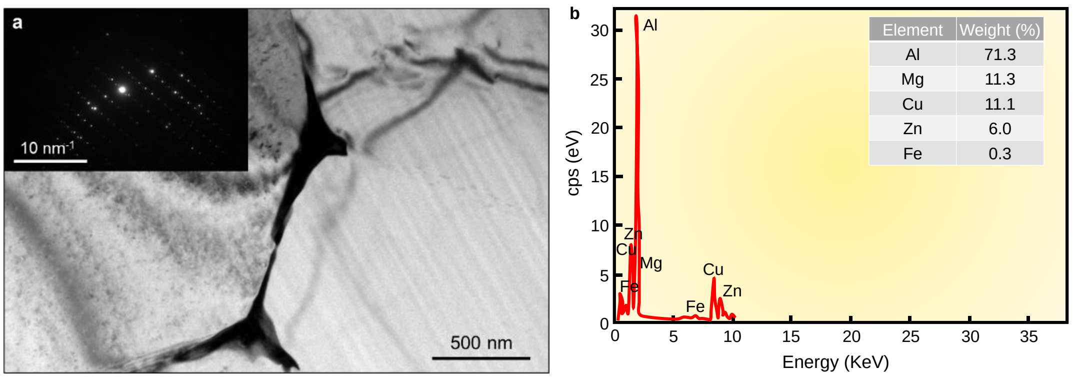

Supplementary Fig. 5: TEM-EDS characterization of the intergranular phase responsible for micro-void nucleation. a, TEM image and SAED. **b**, EDS spectra of the intergranular phase.

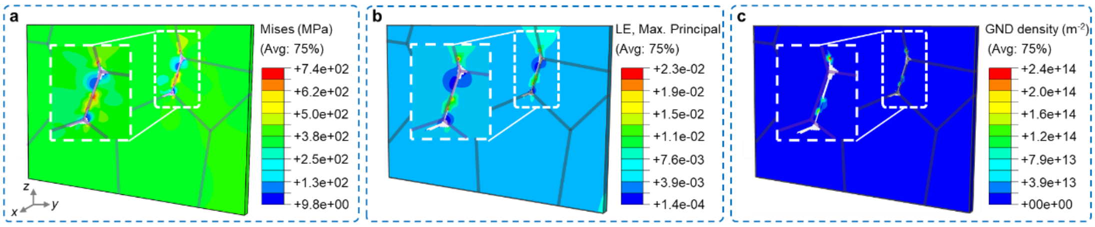

Supplementary Fig. 6: Crystal plasticity finite element simulation showing the deformation across the grains and the intergranular phase (magnified inset). a, von Mises stress. **b**, Logarithmic strain (LE). **c**, Geometrically necessary dislocation (GND) density.

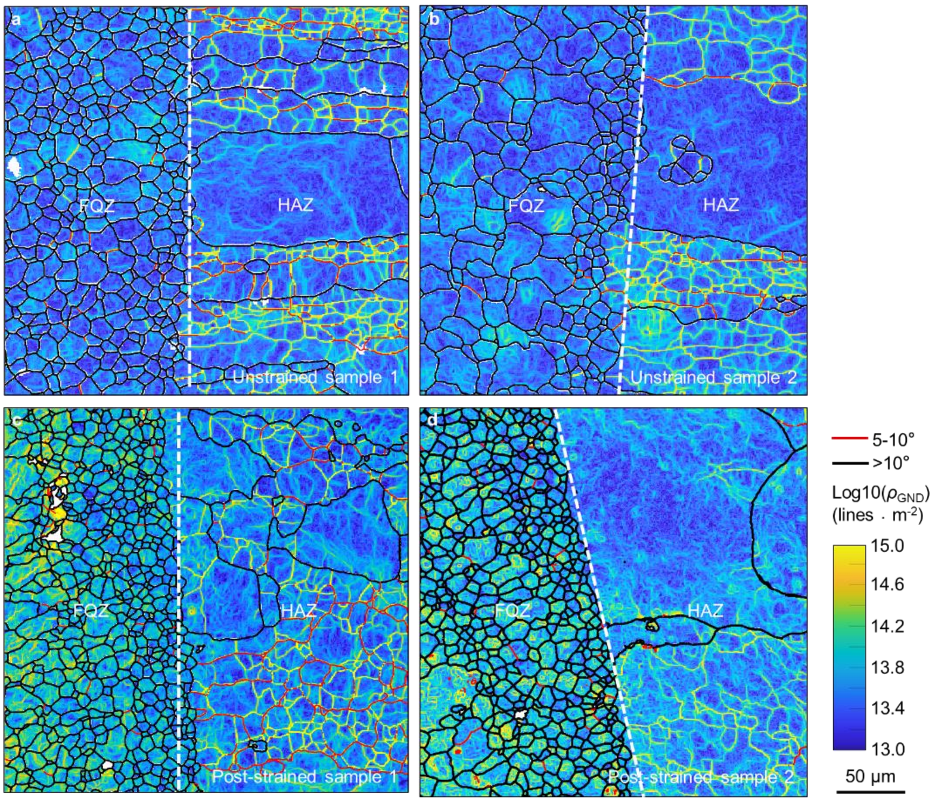

Supplementary Fig. 7: Distribution of the inferred GND density across the cross-section (y-z section) of welded joints. a and **b**, Unstrained samples. **c** and **d**, Post-strained samples.

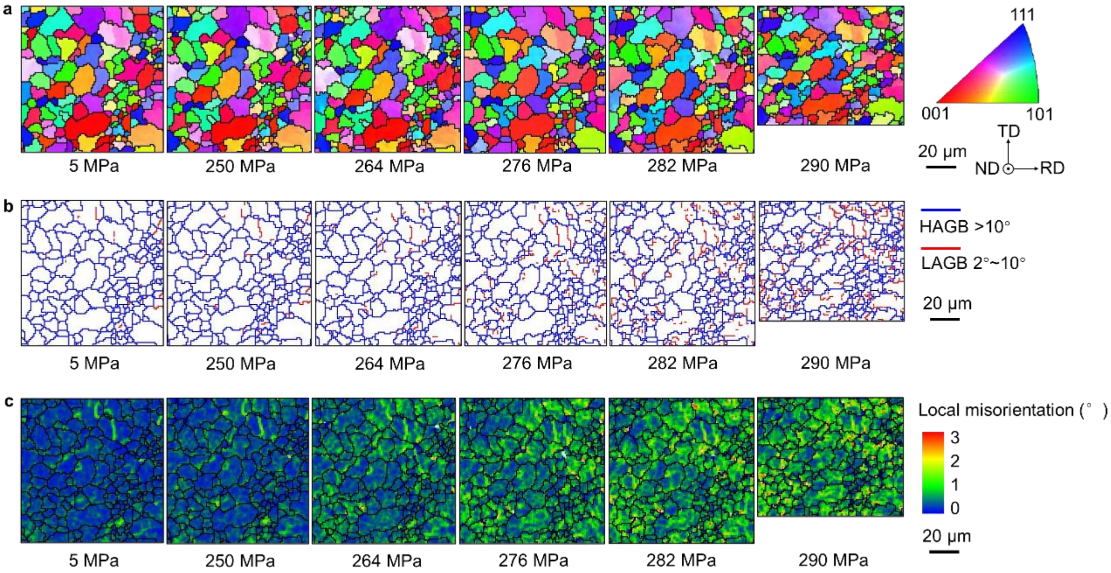

Supplementary Fig. 8: Variation in grain characteristics recorded by EBSD in the FQZ during in situ tensile testing where the loading direction was parallel to the rolling direction. a, Grain orientation. **b**, Grain boundary character. **c**, Local misorientation.

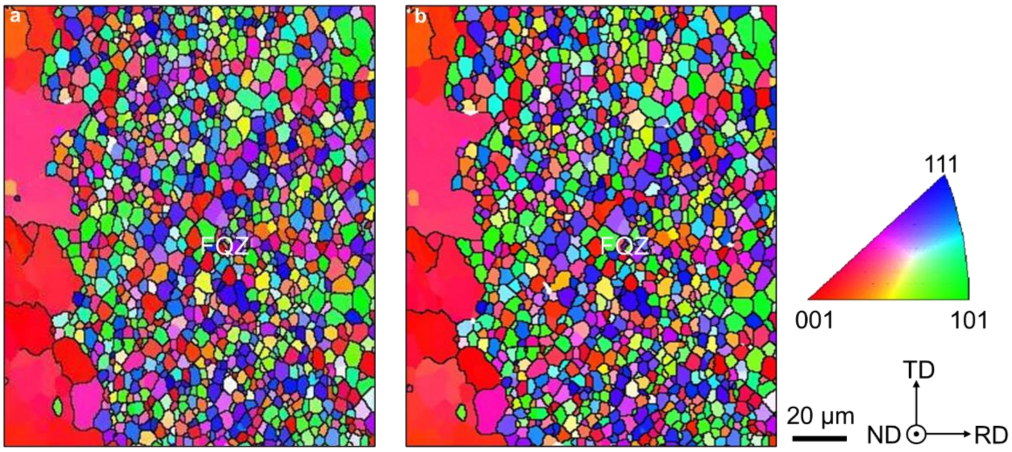

Supplementary Fig. 9: EBSD X IPF maps showing distribution of the FQZ. a, Room temperature. **b,** 470 °C solution heat treatment.

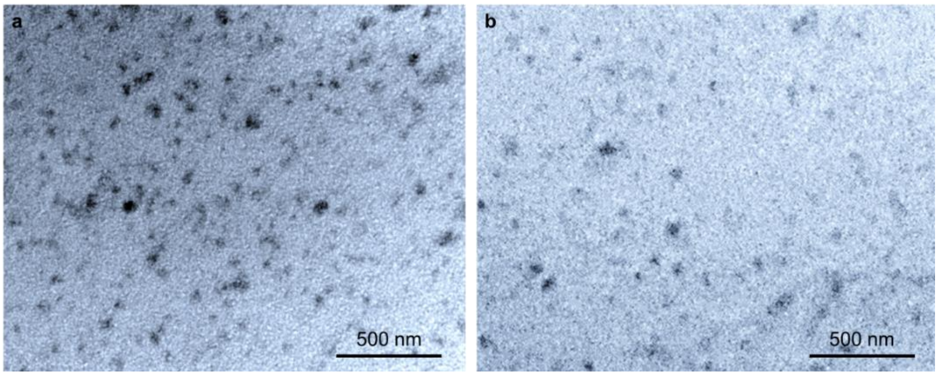

Supplementary Fig. 10: Bright-field TEM images of precipitates in the interior of grains in the FQZ. a, oscillating laser hybrid weld with pulse magnetic field. **b**, conventional laser hybrid weld.

Supplementary References

1. Li, Y., Zhao, Y. Q., Wang, J. Y. & Zhan, X. H. Effect of laser power on the grain morphology and microhardness of dual laser-beam bilateral synchronous welded 2219 aluminium alloy T-joint. *Sci. Technol. Weld. Joi.* **26**, 540-550 (2021).
2. Li, H., Zou, J. S., Yao, J. S. & Peng, H. P. The effect of TIG welding techniques on microstructure, properties and porosity of the welded joint of 2219 aluminum alloy. *J. Alloys Compd*. **727**, 531-539 (2017).
3. Yan, S. H. *et al*. Effect of filling materials on the microstructure and properties of hybrid laser welded Al-Mg-Si alloys joints. *Mater. Des.* **144**, 205-218 (2018).
4. Hu, Y. N. *et al*. Effect of microstructural features on the failure behavior of hybrid laser welded AA7020. *Fatigue Fract. Eng. M.* **41**, 2010-2023 (2018).
5. Qiao, J. N., Lu, J. X. & Wu, S. K. Fatigue cracking characteristics of fiber Laser-VPTIG hybrid butt welded 7N01P-T4 aluminum alloy. *Int. J. Fatigue* **98**, 32-40 (2017).
6. Guo, W. *et al*. Microstructure and mechanical properties of laser welded S960 high strength steel. *Mater. Des.* **85**, 534-548 (2015).

7. Yang, M. H. *et al*. Fine-grain heat affected zone softening of G115/Sanicro25 dissimilar steel welded joints after post-weld heat treatment. *Int. J. Pres. Ves. Pip.* **188**, 104253 (2020).

8. Liu, Y. *et al*. Microstructure evolution of fine-grained heat-affected zone of Gr.92 steel welded joint during creep. *Metall. Mater. Trans. A* **50**, 3080-3090 (2019).
