# Supplementary material for: Inhibiting weld cracking in high-strength aluminium alloys
Source: Nat Commun. 2022 Oct 3;13:5816. doi: 10.1038/s41467-022-33188-x (PMC9530225; doi:10.1038/s41467-022-33188-x)
Supplement: Supplementary file 3 — Description of Additional Supplementary Files [file 41467_2022_33188_MOESM3_ESM.pdf]

Description of Additional Supplementary Files

File Name: Supplementary Movie 1
Description: An animation showing damage development in the weld from crack nucleation through growth to final failure (pores rendered green and cracks yellow). The loading direction is perpendicular to the weld.
